# Genomic investigation of co-targeting tumor immune microenvironment and immune checkpoints in pan-cancer immunotherapy

**DOI:** 10.1038/s41698-020-00136-1

**Published:** 2020-11-13

**Authors:** Xing Huang, Tianyu Tang, Gang Zhang, Zhengtao Hong, Jian Xu, Dipesh Kumar Yadav, Xueli Bai, Tingbo Liang

**Affiliations:** 1 Zhejiang Provincial Key Laboratory of Pancreatic Disease, the First Affiliated Hospital, School of Medicine, Zhejiang University, Hangzhou 310003 Zhejiang, China; 2 Department of Hepatobiliary and Pancreatic Surgery, the First Affiliated Hospital, School of Medicine, Zhejiang University, Hangzhou 310003 Zhejiang, China; 3 Innovation Center for the Study of Pancreatic Diseases, Zhejiang Province, Hangzhou 310003 Zhejiang, China

**Keywords:** Cancer genomics, Cancer microenvironment

## Abstract

Drugs that target immune checkpoints (ICPs) have become the most popular weapons in cancer immunotherapy; however, they are only beneficial for a small fraction of patients. Accumulating evidence suggests that the tumor immune microenvironment (TIME) plays a critical role in anti-cancer immunity. This study aimed to assess the potential merits and feasibility of combinational targeting ICPs and TIME in cancer immunotherapy. A total of 31 cancer type-specific datasets in TCGA were individually collected by the publicly available web servers for multiple bioinformatic analyses of ICPs and TIME factors. GEPIA was used to calculate the prognostic indexes, STRING was used to construct protein–protein interactions, cBioPortal was used for visualization and comparison of genetic alterations, and TISIDB was used to explore the correlation to tumor-infiltrating lymphocytes (TILs). Intriguingly, TIME factors were identified to have more global coverage and prognostic significance across multiple cancer types compared with ICPs, thus offering more general targetability in clinical therapy. Moreover, TIME factors showed interactive potential with ICPs, and genomic alteration of TIME factors coupled with that of ICPs, at least in pancreatic cancer. Furthermore, TIME factors were found to be significantly associated with TILs, including but not limited to pancreatic cancer. Finally, the clinical significance and translational potential of further combination therapies that incorporate both ICP inhibitors and TIME factor-targeted treatments were discussed. Together, TIME factors are promising immunotherapeutic targets, and a combination strategy of TIME factors-targeted therapies with ICP inhibitors may benefit more cancer patients in the future.

## Introduction

The capability of the immune system to recognize and eradicate malignant cells was already identified several decades ago, and dysfunction of the immune system was deemed as one of the hallmarks of cancer development^1^.

Cancer immunotherapies were developed to reactivate the antitumor immune response, thus helping to recognize and eliminate tumors. Their therapeutic efficacy was largely demonstrated by drugs that target immune checkpoints (ICPs), including but not limited to anti-cytotoxic T-lymphocyte-associated protein 4 (CTLA-4) and anti-programmed cell death protein-1 (PD-1)/PD-1 ligand 1 (PD-L1), which significantly improved the prognosis of advanced cancer patients^2–8^. Initial success was achieved in melanoma, following which immune checkpoint blockade (ICB)-based therapeutic strategies have accomplished notable prominence in cancer research and therapy. Dozens of phase III clinical trials have been performed and generally showed a survival benefit of ICB over conventional chemotherapy. Keytruda (i.e., Pembrolizumab, the first PD-1 blocking antibody) was projected to be one of the best-selling drugs in 2020. In fact, ICB represents a specific treatment category that has seen some of the highest FDA and regulatory approvals across many cancer types within a relatively short time—in the past over 5 years based on large randomized trials proving survival benefit even as single agents, whether biomarker-driven or not. Currently, for patients with advanced head and neck squamous cell carcinoma (HNSC), non-small cell lung cancer (NSCLC, squamous and non-squamous carcinoma), melanoma, urothelial and kidney cancers, Merkel cell carcinoma, refractory Hodgkin lymphoma, cancers with high microsatellite instability (MSI; e.g., MSI-high colorectal cancer), and hepatocellular carcinoma, ICB has become a vital part of the standard care; moreover, clinical trials have been initiated to investigate their efficacy for the treatment of additional malignant diseases^2,9–18^.

However, although ICB is frontline therapy for many cancers, increasing numbers of studies have shown that the positive response rate from patients receiving drugs that target ICPs remains quite low in several specific malignancies. Prominent examples are pancreatic cancer^19^, cholangiocarcinoma (CHOL)^20^, and gastric cancer^21^, and this issue has not been resolved to date^22,23^. In the majority of patients, clinical benefits are commonly prevented by acquired tumor resistance and primary tumor refractoriness to ICP-targeting drugs^24,25^. More importantly, clinical decisions to use these drugs, especially dual CTLA-4 and PD-1 blockade, should consider their potential to induce high-grade immune-related adverse events. Concerns over safety-related problems have arisen in connection with multiple cancer therapies, which imposes restrictions on the wide application of ICB^26,27^. Therefore, it is necessary to develop a more effective and safe anticancer immunotherapeutic strategy.

Furthermore, many factors co-contribute to anticancer immunity, and ICPs might only be one type of weapon utilized by tumors to counter attacks from the immune system^28^. Accumulating evidence strongly suggests the tumor immune microenvironment (TIME) as the battlefield between tumor cells and the immune system. Consequently, the TIME also plays a significant role in tumor immune surveillance and immunological evasion, and thus exerts an enormous influence on the final outcome of cancer immunotherapy^16,29,30^. Therefore, to further improve the therapeutic precision and limit the side-effects of ICB-based therapies, this study combined The Cancer Genome Atlas (TCGA) and several other open-access genomic databases to analyze the defects of ICB treatment. The potential for targeting TIME factors in anticancer immunotherapy was highlighted, and a combination strategy of co-targeting ICPs and TIME factors was further discussed to increase tumor immunogenicity, favor intra-tumoral T-cell infiltration, and enhance ICB efficacy. In other words, this study suggested the significance of the incorporation of the targeting of TIME factors into anticancer immunotherapy. It also indicated the feasibility of combined treatment to boost a controllable anticancer immune response to overcome immunotherapeutic resistance in clinical applications.

## Results

### The prognostic landscape of ICPs across multiple cancer types

**Fig. 1:**
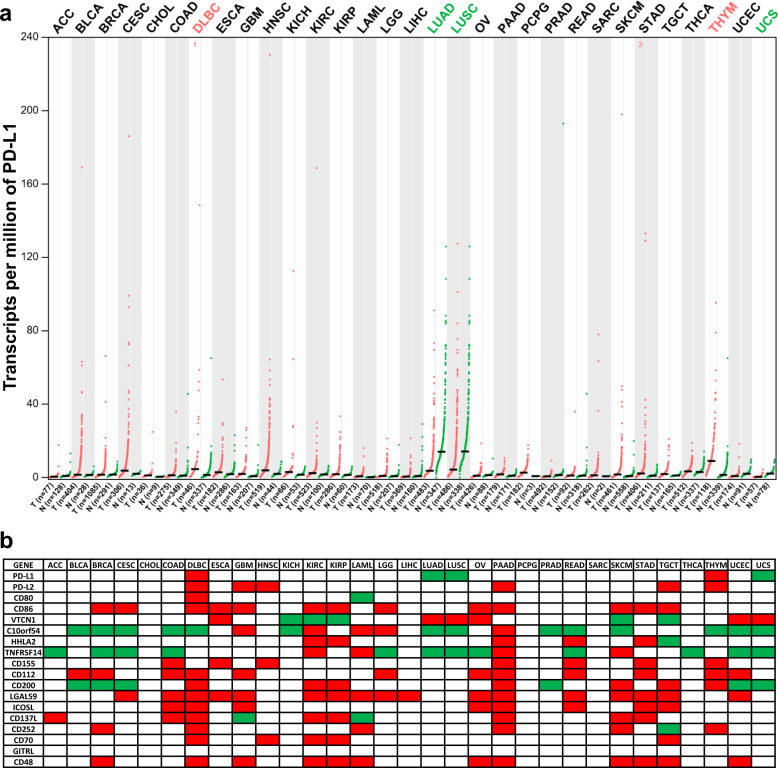
Expression profile of ICPs across multiple cancer types. **a** Expression profile of PD-L1 in multiple cancer types. GEPIA generated dot plots profiling the tissue-wise expression patterns of PD-L1 across multiple cancer types (TCGA tumor) and paired normal tissue samples (TCGA normal + GTEx normal). Each dot represents the individual expression of a distinct tumor or normal sample. **b** Summary of expression profiles of ICPs in multiple cancer types. Differential expression profiles of ICPs were individually analyzed using GEPIA and subsequently integrated together. Red blocks represent the ICPs upregulated in the tumor, green blocks represent the ICPs downregulated in the tumor, and blank blocks indicate the ones are not significantly differentially expressed between tumoral and normal tissues. ANOVA method was used for differential gene expression analysis, and genes with higher |log2FC| values (> 1) and lower *q* values (< 0.01) were considered differentially expressed genes.

**Fig. 2:**
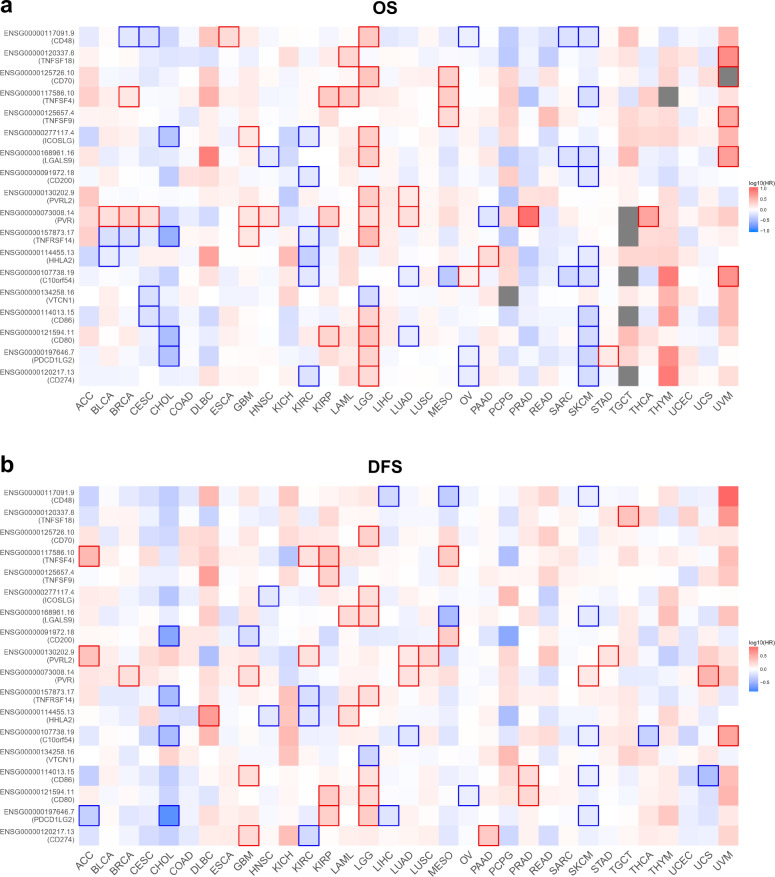
Survival contribution of ICPs across multiple cancer types. **a** Contribution of ICPs to OS in multiple cancer types. GEPIA generated the Kaplan–Meier OS map comparing the groups with different expression levels of ICPs in multiple cancer types (TCGA tumors). **b** Contribution of ICPs to DFS in multiple cancer types. GEPIA generates the Kaplan–Meier DFS map comparing the groups with different expression levels of ICPs in multiple cancer types (TCGA tumors). Red blocks represent ICPs unfavorable to survival, blue blocks represent ICPs favorable to survival, and the ones with outer wireframe indicate significant influence. Mantel–Cox test was used for the hypothesis tests, and the Cox proportional hazard ratio was included in the survival plots. A *p* value < 0.05 was considered to be statistically significant.

To date, PD-L1, also known as CD274 or B7-H1, is one of the most important and representative ICPs. Multiple malignancies, employ an immune shield by expressing PD-L1 to attack the immune system and avoid elimination^2,3^. To further clarify the significance of ICP targeting, this study first chose PD-L1 as an example of a critical target of ICB to conduct globally prognostic analyses via gene expression profiling interactive analysis (GEPIA). To analyze the targetability of PD-L1, the expression profile of PD-L1 was investigated in the following 31 major cancers in the TCGA database: adrenocortical carcinoma (ACC), bladder urothelial carcinoma (BLCA), breast invasive carcinoma (BRCA), cervical squamous cell carcinoma and endocervical adenocarcinoma (CESC), CHOL, colon adenocarcinoma (COAD), lymphoid neoplasm diffuse large B-cell lymphoma (DLBC), esophageal carcinoma (ESCA), glioblastoma multiforme (GBM), HNSC, kidney chromophobe (KICH), kidney renal clear cell carcinoma (KIRC), kidney renal papillary cell carcinoma (KIRP), acute myeloid leukemia (LAML), rain lower grade glioma (LGG), liver hepatocellular carcinoma (LIHC), ung adenocarcinoma (LUAD), lung squamous cell carcinoma (LUSC), ovarian serous cystadenocarcinoma (OV), pancreatic adenocarcinoma (PAAD), pheochromocytoma and paraganglioma (PCPG), prostate adenocarcinoma (PRAD), rectum adenocarcinoma (READ), sarcoma (SARC), skin cutaneous melanoma (SKCM), stomach adenocarcinoma (STAD), testicular germ cell tumors (TGCT), thyroid carcinoma (THCA), thymoma (THYM), terine corpus endometrial carcinoma (UCEC), and uterine carcinosarcoma (UCS). Differential expression analysis showed that compared with normal tissue, the expression levels of PD-L1 were significantly upregulated in DLBC and THYM and downregulated in LUAD, LUSC, and USC (Fig. 1a). Further analysis indicated significant deregulation of the expression levels of other different ICPs in the majority of malignancies (Fig. 1b). Furthermore, survival analysis showed that the expression levels of ICPs were significantly associated with overall survival (OS) (Fig. 2a) and disease-free survival (DFS) (Fig. 2b). Malignancies can be divided into three major categories according to the results of differential expression and survival analysis: (1) No ICP was identified that was significantly deregulated (e.g., CHOL and PCPG). (2) ICPs were found to be significantly deregulated but did not influence prognosis (e.g., COAD and THYM). (3) ICPs were found to be deregulated and significantly influenced prognosis (e.g., LGG and KIRC).

### The prognostic landscape of TIME factors across multiple cancer types

**Fig. 3:**
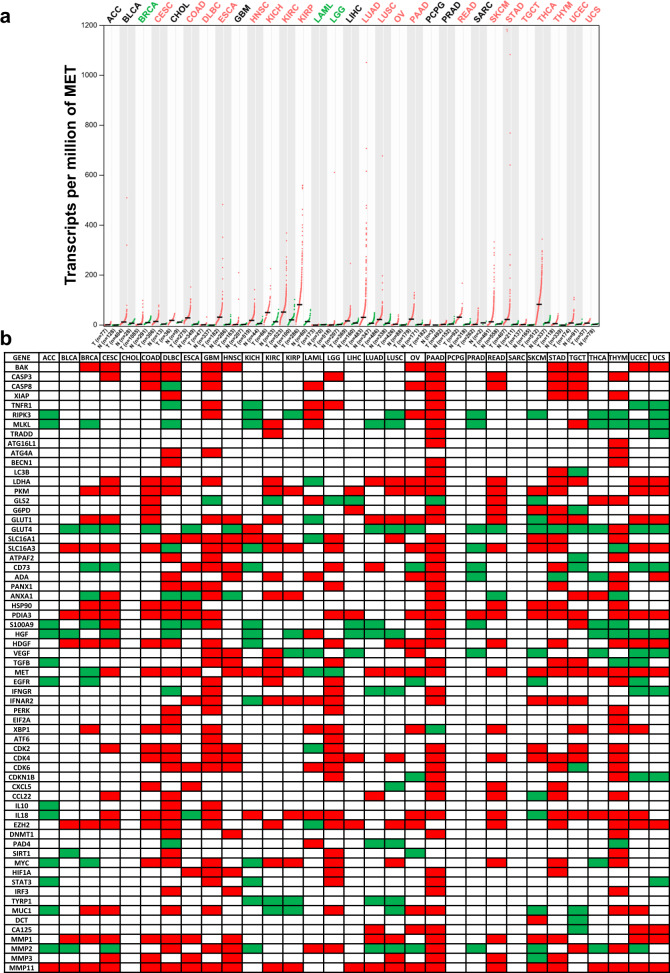
Expression profile of TIME factors across multiple cancer types. **a** Expression profile of MET in multiple cancer types. GEPIA generated dot plots profiling the tissue-wise expression patterns of MET across multiple cancer types (TCGA tumor) and paired normal tissue samples (TCGA normal + GTEx normal). Each dot represents the individual expression of a distinct tumor or normal sample. **b** Summary of expression profiles of TIME factors in multiple cancer types. Differential expression profiles of TIME factors were individually analyzed using GEPIA and subsequently integrated together. Red blocks represent the TIME factors upregulated in the tumor, green blocks represent the TIME factors downregulated in the tumor, and blank blocks indicate the ones are not significantly differentially expressed between tumoral and normal tissues. The ANOVA method was used for differential gene expression analysis, and genes with higher |log2FC| values (> 1) and lower *q* values (< 0.01) were considered differentially expressed genes.

**Fig. 4:**
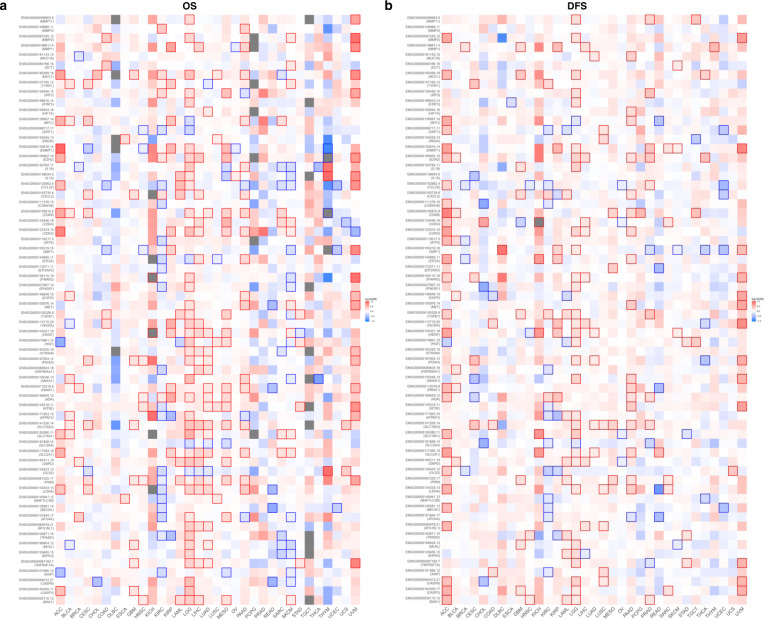
Survival contribution of TIME factors across multiple cancer types. **a** Contribution of TIME factors to OS in multiple cancer types. GEPIA generated the Kaplan–Meier OS map comparing the groups with different expression levels of TIME factors in multiple cancer types (TCGA tumor). **b** Contribution of TIME factors to DFS in multiple cancer types. GEPIA generates the Kaplan–Meier DFS map comparing the groups with different expression levels of TIME factors in multiple cancer types (TCGA tumor). Red blocks represent TIME factors unfavorable to survival, blue blocks represent TIME factors favorable to survival, and the ones with outer wireframe indicate significant influence. Mantel–Cox test was used for the hypothesis tests, and the Cox proportional hazard ratio was included in the survival plots. A *p* value <0.05 was considered to be statistically significant.

Considering the expression spectrum and prognostic uncertainty of ICPs in cancer, the widespread application of ICP inhibitors is perhaps unrealistic. ICB is not sufficient for cancer immunotherapy. As mentioned before, TIME is another key determinant for cancer therapeutic efficacy, and the significance of TIME for the optimization of cancer therapeutic efficacy should not be entirely neglected. The influence of TIME factors was investigated through differential expression analysis and survival analysis using GEPIA. Firstly, MET (HGF receptor, traditional receptor tyrosine kinase but with a novel regulatory function in cancer immunity^31–33^) was chosen as a representative TIME factor. Compared with normal tissue, the expression level of MET was downregulated in BRCA, LAML, and LGG and upregulated in 20 types of cancers including CESC, COAD, and PAAD (Fig. 3a). Further differential expression analysis indicated that TIME factors were significantly deregulated in the majority of malignancies (Fig. 3b). In addition, survival analysis showed that the expression levels of TIME factors were significantly associated with OS (Fig. 4a) and DFS (Fig. 4b). Malignancies can be divided into three major categories according to the results of differential expression and survival analysis: (1) TIME factors that were deregulated and had a significant influence on prognosis (e.g., LGG and KIRC), which suggests that they are potentially promising targets for cancer therapy and that targeting TIME regulators may effectively benefit cancer patients. (2) TIME factors that were deregulated but did not influence prognosis (e.g., DLBC and PRAD), suggesting that they may have minimal impact on and may thus not be appropriate targets for such cancer types. (3) No TIME factors were significantly deregulated (e.g., CHOL, PCPG, and SARC), indicating that these three types of cancers may be TIME-factor independent.

### Interaction between ICPs and TIME factors

**Fig. 5:**
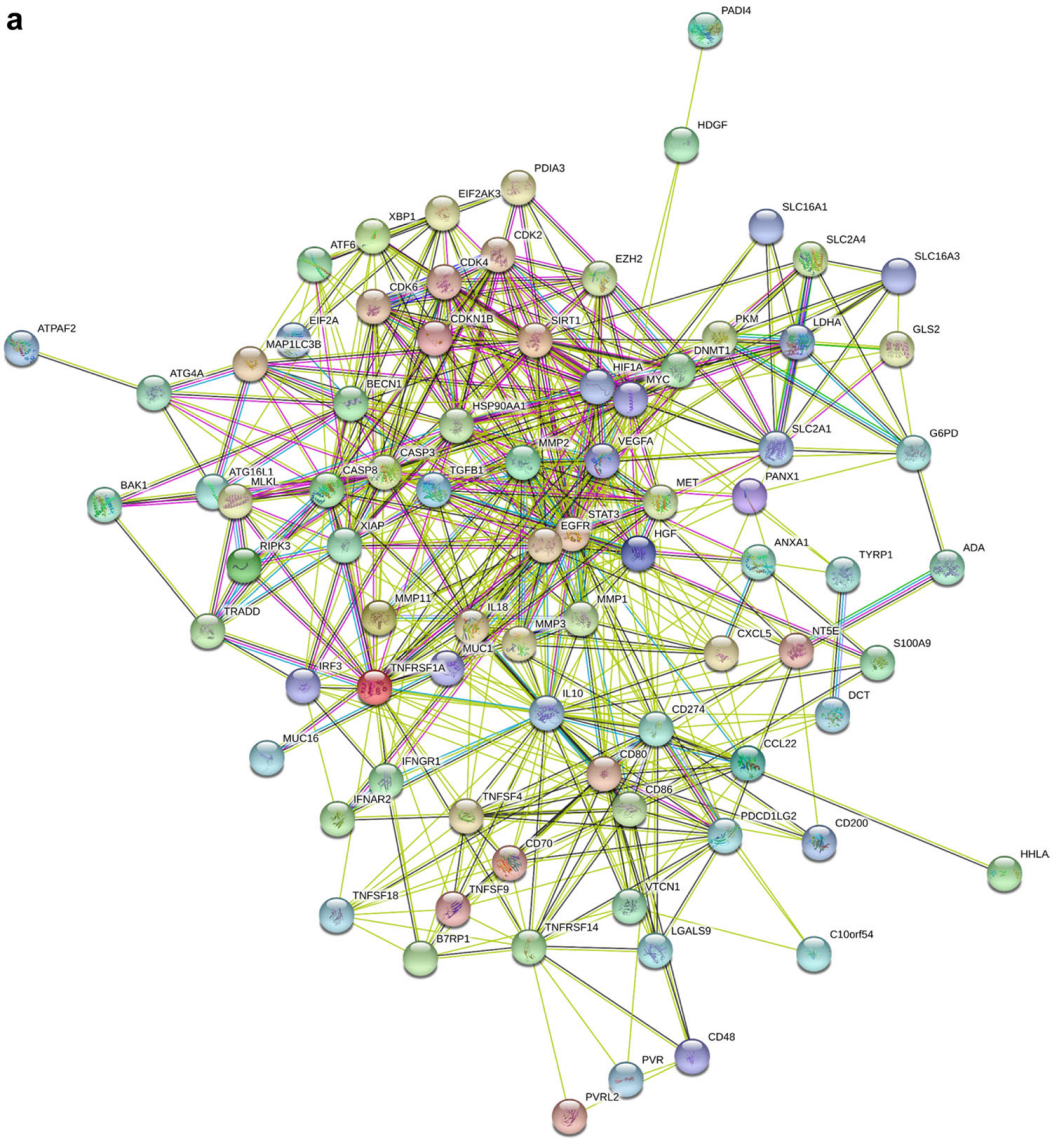
Interaction network between ICPs and TIME factors. **a** Landscape of interplay of ICPs and TIME factors. STRING generated known and predicted protein–protein interactions between ICPs and TIME factors, including direct (physical) binding and indirect (functional) association, stemmed from interactions aggregated from primary databases, knowledge transfer between organisms, and computational prediction. Network nodes represent proteins, and edges represent protein–protein associations. The combined scores indicating the confidence in the interaction rank from 0 to 1, with 1 being the highest possible confidence.

**Fig. 6:**
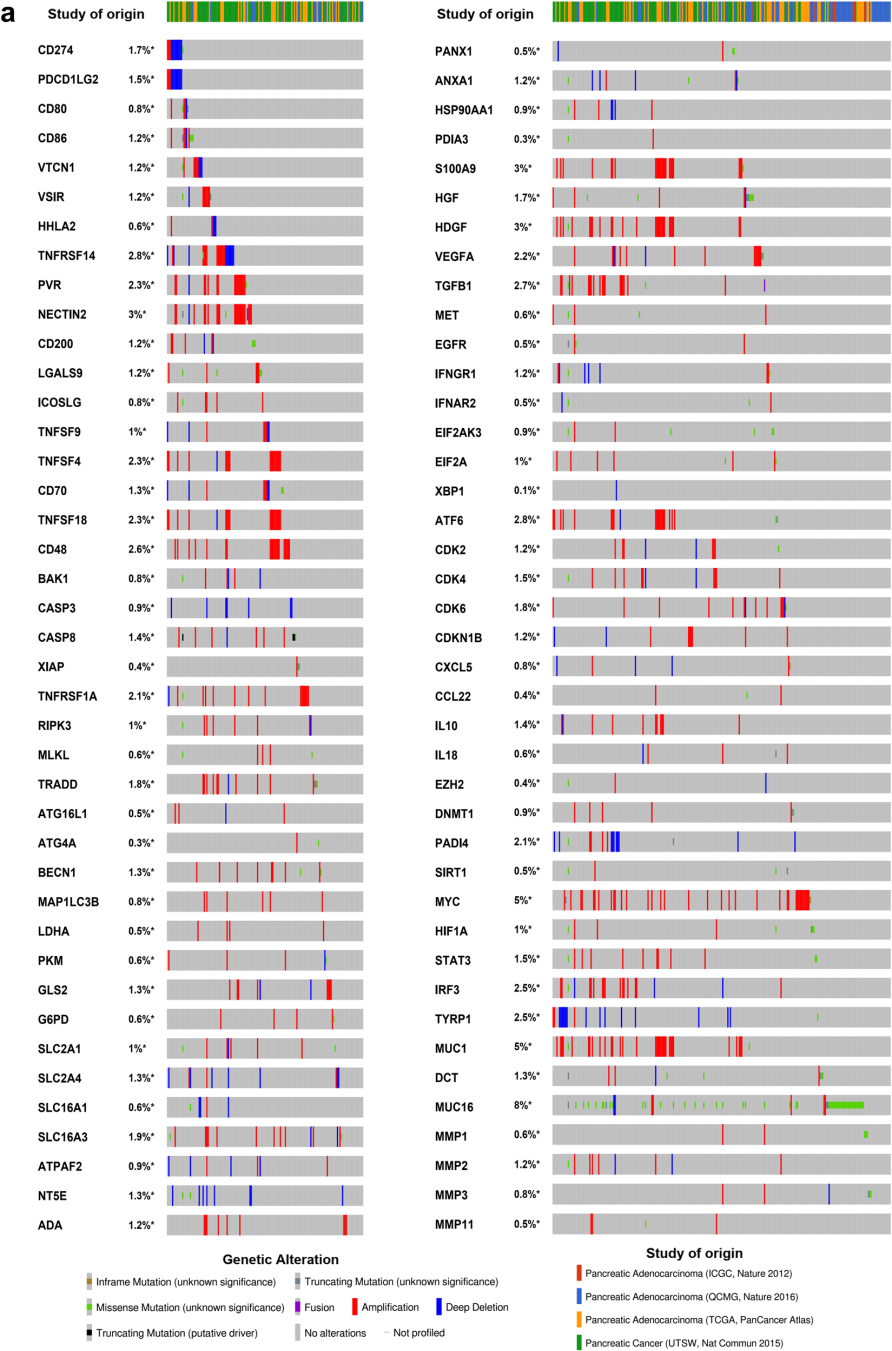
Genetic correlation between alterations of ICPs and TIME factors in pancreatic cancer. **a** Landscape of genetic alterations of ICPs and TIME factors in pancreatic cancer. Compact visualization of cases originated from four studies with multiple genetic alterations of ICPs and TIME factors were individually shown by cBioPortal as indicated, including the cases with inframe mutation, missense mutation, truncating mutation, fusion, amplification, deep deletion or/and no alterations, and not profiled ones.

**Fig. 7:**
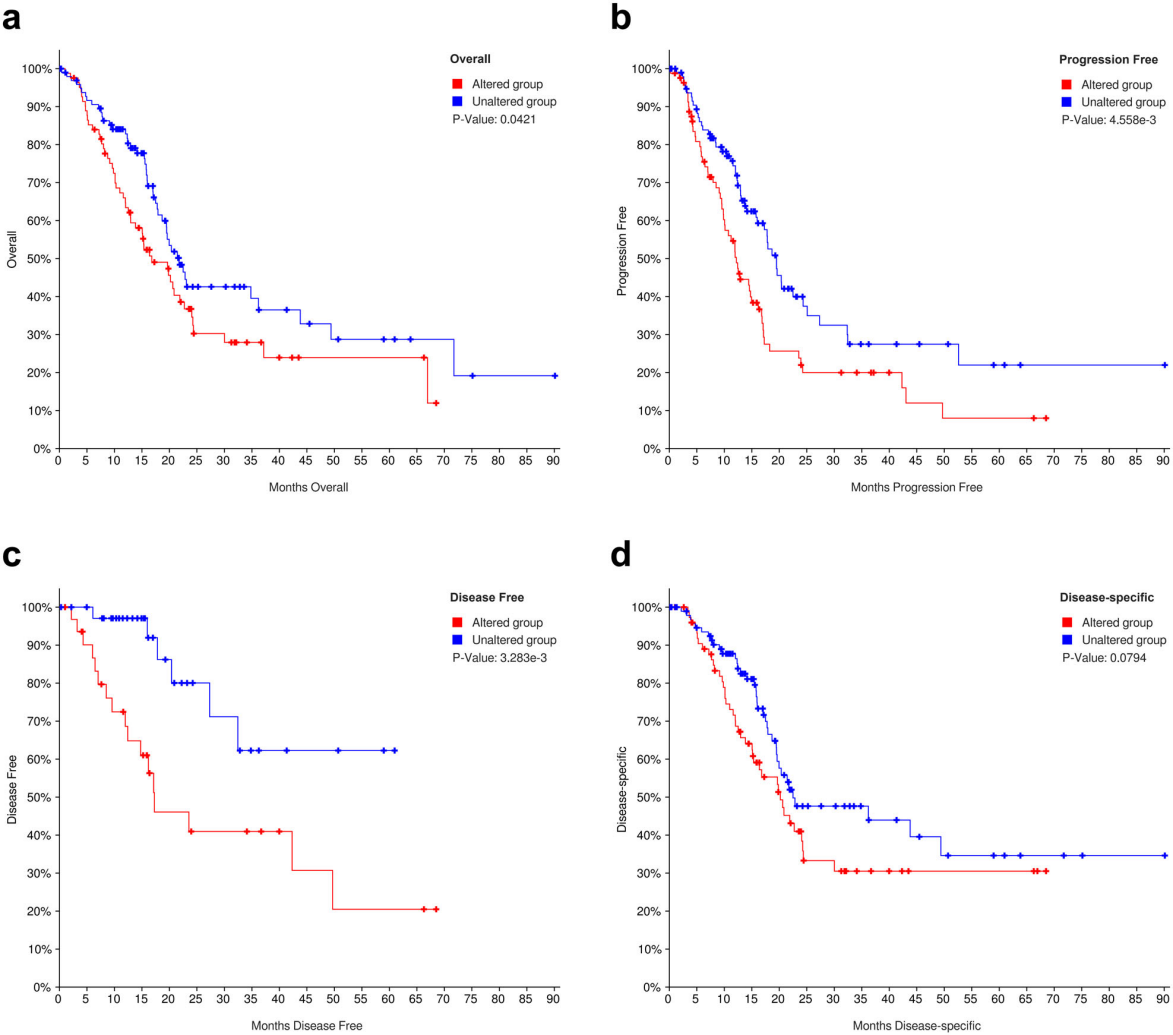
Multiple survival analyses of integrated alterations of ICPs and TIME factors in pancreatic cancer. **a** Contribution of integrated alterations of ICPs and TIME factors to OS in pancreatic cancer. Patients with or without different alterations of ICPs and TIME factors were individually collected and subjected to OS analysis. The time period covers overall patient survival status. **b** Contribution of integrated alterations of ICPs and TIME factors to PFS in pancreatic cancer. Patients with or without different alterations of ICPs and TIME factors were individually collected and subjected to PFS analysis. The time period covers progression-free status. **c** Contribution of integrated alterations of ICPs and TIME factors to DFS in pancreatic cancer. Patients with or without different alterations of ICPs and TIME factors were individually collected and subjected to DFS analysis. The time period covers disease-free status since the initial treatment. **d** Contribution of integrated alterations of ICPs and TIME factors to DSS in pancreatic cancer. Patients with or without different alterations of ICPs and TIME factors were individually collected and subjected to DSS analysis. The time period usually begins at the time of diagnosis or at the start of treatment and ends at the time of death. ICPs and TIME factors are altered in 252 (30%) of queried samples. Red curves represent the altered groups, and blue curves represent the unaltered groups. A log-rank test was used for the hypothesis test, and a *p* value < 0.05 was considered to be statistically significant.

Since both ICPs and TIME factors are critical for the prognosis of cancer patients, the next step investigated the existence of potential interplays between ICPs and TIME factors. In general, a strong protein–protein interaction between ICPs and TIME factors was observed using the Search Tool for Recurring Instances of Neighboring Genes (STRING) (Fig. 5a). This included direct (physical) binding and indirect (functional) association, originating from interactions aggregated from primary databases. Since almost all ICPs and TIME factors were upregulated in PAAD, pancreatic adenocarcinoma was chosen for further detailed investigation, particularly the assessment of the potential combined targetability of ICPs and TIME factors. The global landscape of the genomic alteration of ICPs and TIME factors in pancreatic cancer was visualized using cBioPortal. This landscape included inframe mutation, missense mutation, truncating mutating, fusion, amplification, deep deletion, and no alterations (Fig. 6a). The detailed correlation between each TIME factor and ICP was individually analyzed, and statistically significant relationship was presented in Supplementary Table 1. For example, MUC1, a cell surface TIME factor found on epithelial cells, is found associated with HDGF where they share 26 variants in 769 patient samples. The genomic alterations of TIME factors showed general co-occurrence rather than mutual exclusivity with ICPs. In fact, a total of 591 significant associations between two genes among TIME factors and ICPs were observed in this analysis, all of which showed co-occurrence but not mutual exclusivity. Furthermore, integrated prognostic analyses of OS (Fig. 7a), progression-free survival (PFS) (Fig. 7b), DFS (Fig. 7c), and disease-specific survival (DSS) (Fig. 7d) indicated that integrated genomic alterations of TIME factors and ICPs were significantly unfavorable for multiple prognoses of patients with pancreatic cancer.

### Associations between ICPs, TIME factors, ICD mediators, and cancer immunity

Given the complexity of the interaction between TIME factors and ICPs and their enormous influence on the tumor immune suppressive microenvironment, combination therapy might be indicated to efficiently reinvigorate the immune system against tumors. However, directly targeting multiple factors in a simple and feasible way still remains difficult. Over the past 10 years, ever-growing evidence suggests the induction of immunogenic cell death (ICD) for immune system activation as an effective method for cancer immunotherapy^22,27,34^. ICD is capable to elicit antitumor immunity via dead cell-associated antigens, and the molecular properties of ICD have been verified to largely overlap with TIME factors. Exposure of calreticulin (CALR, as an “eat me” signal belonging to the “DAMPs” module and regulated by the “ER stress” module of TIME factors), secretion of adenosine triphosphate (ATP, as a “come to me” signal belonging to the “ATP homeostasis” module and regulated by the “Autophagy” module of TIME factors), the release of high mobility group box 1 (HMGB1, as an “activate you” signal belonging to the “DAMPs” module and regulated by the “Necrosis” module of TIME factors), autocrine of type I interferon (IFN I, as a “stimulate you” signal belonging to the “Cytokine” module and regulated by the “Trans-factor” module of TIME factors), and export of annexin A1 (ANXA1, as a “find me” signal belonging to the “DAMPS” module and regulated by the “Necrosis” module of TIME factors) from dying cancer cells have been individually identified as the five key hallmarks in processing ICD^35^. At the beginning, the secreted ATP favors the recruitment and activation of antigen-presenting cells (APCs) by P2RY2 and P2RX7^36^. The exported ANXA1 guides the homing and juxtaposition of APCs to dying cells by FPR1^37^. Then, exposed CALR promotes the engulfment of dying cells and antigen uptake by LRP1^38^. Furthermore, the released HMGB1 stimulates the synthesis of pro-inflammatory factors, APC maturation, and presentation of tumor antigens by TLR4^39^. Finally, autonomous type I IFN increases CXCL10 secretion and T-cell recruitment, thus exerting antitumor effects^40,41^. Of note, such hallmarks of ICD can be triggered by multifarious cellular stress, including ER stress-caused CALR exposure, autophagy-induced ATP secretion, secondary necrosis-engendered HMGB1, and ANXA1 release, as well as infectious pathogens that stimulate autonomous type I IFN^36,41–47^. Hence, widely applicable inducers of ER stress, autophagy, necroptosis, or viral mimicry have been employed as ICD activators^15,34,48–53^.

**Fig. 8:**
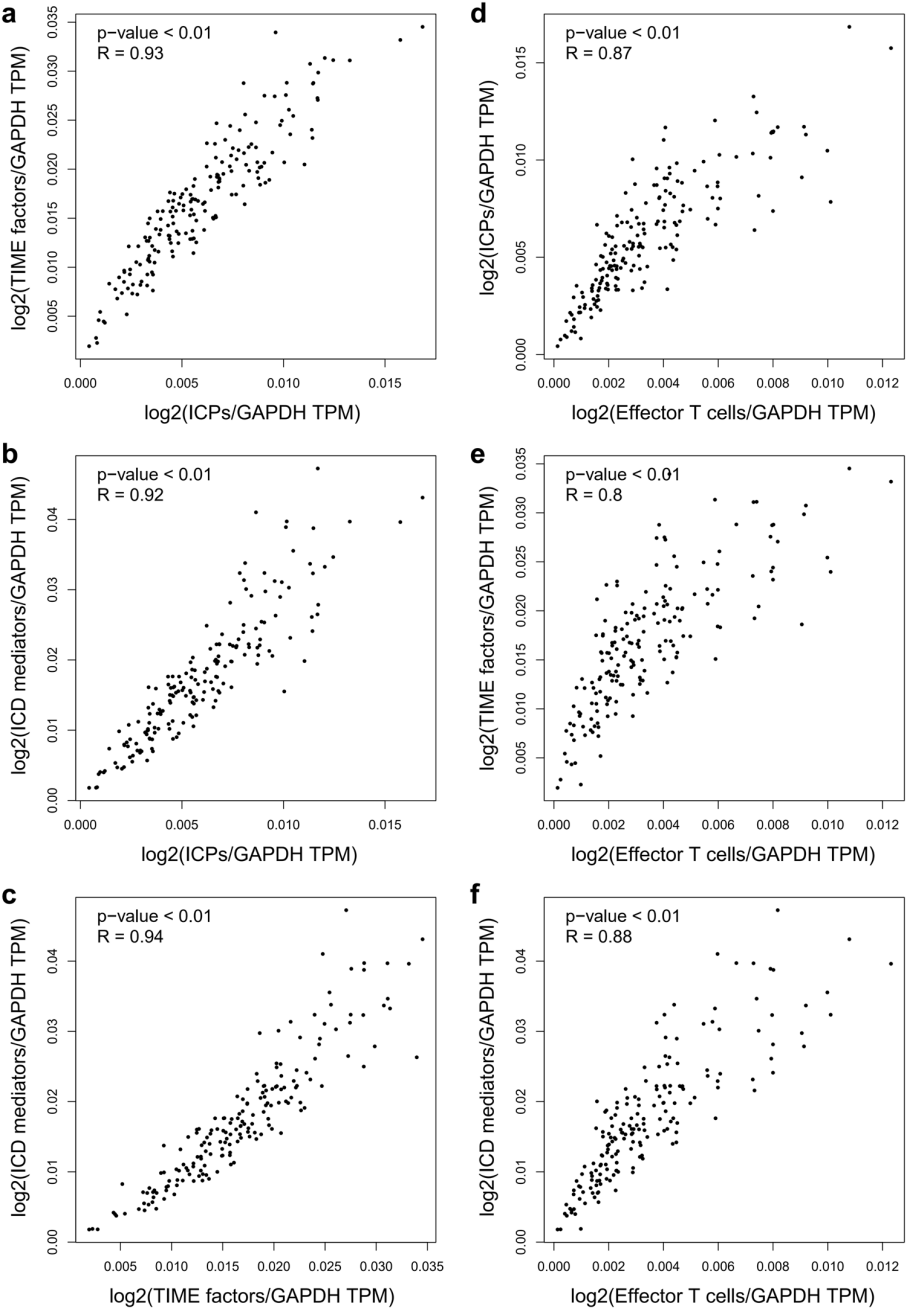
Potential relevance between ICPs, TIME factors, ICD mediators, and effector T-cell signatures in pancreatic cancer. **a** Correlation of ICPs and TIME factors in pancreatic cancer. **b** Correlation of ICPs and ICD mediators in pancreatic cancer. **c** Correlation of TIME factors and ICD mediators in pancreatic cancer. **d** Correlation of ICPs and effector T-cell signatures in pancreatic cancer. **e** Correlation of TIME factors and effector T-cell signatures in pancreatic cancer. **f** Correlation of ICD mediators and effector T-cell signatures in pancreatic cancer. GEPIA generated the pair-wise gene expression correlations between two lists of signature genes in pancreatic cancer (TCGA tumor), using the Spearman method after normalized by GAPDH. Each point represents an independent case. The non-log scale was used for calculation and the log-scale axis was used for visualization. The detailed *p* value and *R* were individually presented as indicated in each panel, and a *p* value < 0.05 was considered statistically significant.

**Fig. 9:**
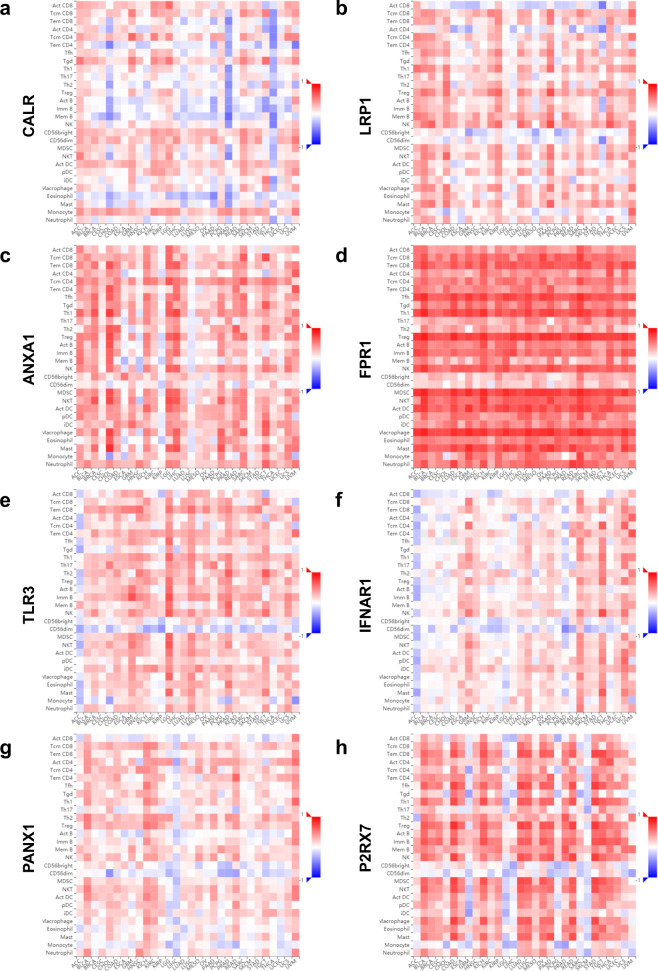
Immunological influence of ICD mediators on TILs across multiple cancer types. **a** Correlations between CALR and TILs in multiple cancer types. **b** Correlations between LRP1 and TILs in multiple cancer types. **c** Correlations between ANXA1 and TILs in multiple cancer types. **d** Correlations between FPR1 and TILs in multiple cancer types. **e** Correlations between TLR3 and TILs in multiple cancer types. **f** Correlations between IFNAR1 and TILs in multiple cancer types. **g** Correlations between PANX1 and TILs in multiple cancer types. **h** Correlations between P2RX7 and TILs in multiple cancer types. TISIDB generated spearman correlations between expression of ICD mediators and an abundance of TILs across multiple cancers (TCGA tumor). The immune-related signatures of 28 TIL types were collected from Charoentong’s study. For each cancer type, the relative abundances of TILs were inferred by using gene set variation analysis based on gene expression profile. Each correlation between an ICD mediator and a distinct TIL in an individual cancer type was integrated into the indicated heatmap.

Thus, the correlations between ICD mediators, TIME factors, ICPs, and effective T-cell signatures were further investigated in pancreatic cancer by GEPIA. First, significant correlations were identified between TIME factors and ICPs (*R* = 0.93, *P* < 0.01) (Fig. 8a), ICD mediators and ICPs (*R* = 0.92, *P* < 0.01) (Fig. 8b), as well as ICD mediators and TIME factors (*R* = 0.94, *P* < 0.01) (Fig. 8c). Furthermore, significant associations were also identified between ICPs and effective T-cell signatures (CX3CR1, FGFBP2, and FCGR3A) (*R* = 0.87, *P* < 0.01) **(**Fig. 8d), TIME factors and effective T-cell signatures (*R* = 0.80, *P* < 0.01) (Fig. 8e), as well as ICD mediators and effective T-cell signatures (*R* = 0.88, *P* < 0.01) (Fig. 8f). To further confirm the correlation of ICD with TILs and its potential influence, eight representative ICD mediators were chosen and correlation analysis was conducted in multiple cancer types using the Tumor and Immune System Interaction Database (TISIDB). The resulting heatmap showed that in the majority of malignancies, TILs were significantly correlated with multiple representative ICD mediators, such as CALR (Fig. 9a), LRP1 (Fig. 9b), ANXA1 (Fig. 9c), FPR1 (Fig. 9d), TLR3 (Fig. 9e), IFNAR1 (Fig. 9f), PANX1 (Fig. 9g), and P2RX7 (Fig. 9h).

## Discussion

To date, ICP inhibitors have become very successful anti-cancer weapons. Dozens of clinical trials have demonstrated the ability of ICP inhibitors to achieve durable objective responses in advanced patients^9,10,13,54^. These encouraging results strongly promote enthusiasm for immunotherapy and underscore a basic fact that at least in a subset of cancer patients, the suppressed immune system can still recognize tumor cells and be reactivated against these if sufficient co-stimulatory signals are delivered. However, the high proportion of acquired resistance and primary refractoriness to ICPs in several specific cancers (e.g., pancreatic cancer) need to be considered. Therefore, the application of ICP inhibitors perhaps is not sufficient to maximize the benefit of cancer immunotherapy. In other words, it is time to incorporate ICP inhibitors into more effective combination therapies. Here, our results revealed the prognostic significance of TIME factors in multiple cancers and their close relationship with ICPs, implicating tumors use both ICPs and TIME factors to escape from immune surveillance. And thus, we proposed that TIME factors are promising cancer immunotherapeutic targets, and targeting TIME factors should be prioritized for combination with ICP inhibitors. This combination strategy may significantly extend cancer immunotherapy and improve its clinical benefit. However, how and in which circumstances TIME factors-targeted therapy should be combined with ICB treatment still need to be addressed case by case in the future because each individual patient has a unique profile of ICPs and TIME factors. Moreover, further study is needed to elucidate the underlying mechanism contributing to the response to immunotherapy in varying patients.

Previous reports associated PD-L1 overexpression with poor prognosis in various cancers^55–57^; however, contradictory results were reported for breast cancer, HNSC, renal cell carcinoma, and upper tract urothelial carcinoma^58–61^. Moreover, PD-L1 expression was upregulated by cancer cells when the tumor was infiltrated with active T cells and in response to the production of interferon-gamma (IFNG); therefore, high PD-L1 expression might indicate a preexisting T-cell response^62^. Interestingly, tumor-infiltrating immune cells might have increased sensitivity to IFNG and upregulate PD-L1 to suppress a preexisting T-cell response prior to treatment. Kowanetz et al. reported more prevalent PD-L1 expression on immune cells and proposed that this was associated with IFN-γ-induced adaptive regulation together with tumor-infiltrating effector T cells and lymphocytes. Although exceptions were occasionally observed in clinical practice, results indicated that patients with tumors that show high PD-L1 expression generally represent a population with an increased likelihood of responding to ICB^13,18,63^. Disrupted epigenetics of the gene encoding PD-L1 (*CD247*) resulted in high PD-L1 expression in tumor cells, which was associated with poor immune infiltration, sclerotic/desmoplastic stroma, and mesenchymal molecular features^64^. As a result, anti-PD1/PD-L1 has become one of the most successful immune therapies, and the expression of PD-L1 in tumor cells was identified as a crucial biomarker for the efficacy of ICP therapy^65^. Therefore, we first chose PD-L1 as an example of a critical target of ICB to conduct globally prognostic analyses via GEPIA.

In addition to the expression level of ICPs, TIME-related markers should also be further considered for a better outcome and safety. For instance, cancer cells can alter the outside microenvironment through characteristic metabolic patterns (e.g., Warburg effect and glutaminolysis), thus inactivating cancer-infiltrating immune cells^66–71^. Moreover, communication between cancer and surrounding stromal cells has been found to be involved in cancer immunologic tolerance and escape, in addition to supporting cancer cell growth and metastasis^65,72–76^. In addition, cancer antigens can be directly modified by glycosylation or cleavaged by extracellular matrix metalloproteinases (MMPs), to avoid recognition by the immune system^77–80^. When attacked by cytotoxic T lymphocytes, cancer cells are able to utilize IFNG in TIME to activate their own signaling pathway related to signal transducers and activators of transcription (STATs), which in turn upregulates PD-L1 expression and suppresses incoming immune attacks^14,81–83^. In summary, to avoid elimination by the immune system, cancer cells succeed in evolving a variety of ways to regulate their surrounding TIME, thus interfering with antitumor immunity or ICB-caused immunotherapeutic stresses. Therefore, even in ICP-amplified cases, many “tunnels” still leave cancer cells capable to escape from or at least survive immune surveillance and therapy. This ultimately results in immunological resistance and poor prognosis.

The high-throughput database provided valuable information toward a deeper understanding of the underlying mechanism of the anticancer efficacy of the immune system. Our results indicated the prevalence of deregulation of both ICPs and TIME factors in varying types of malignancies, which exerted enormous influence on the prognosis of patients. While closely correlated with ICPs, deregulation and genomic alteration of TIME factors greatly affected the antitumor immune function. These results indicated both TIME and ICPs as two insidious weapons used by tumors to escape immune surveillance. As mentioned before, in contrast to direct immune-mediation caused by ICPs, it seems that TIME tends to exert an indirect suppressive effect on the immune system. Of note, the demarcation line between ICPs and TIME factors remains unclear. For instance, CD73 and CD39, identified as ATP homeostasis factors in this study, have also been identified as potential ICPs. Moreover, tumors can utilize ICPs to regulate their surrounding TIME, thus avoiding elimination by the immune system. At least in a fraction of patients, the observed response of ICP inhibitors may result from the capability of these therapies to simultaneously reshape TIME. However, instead of identifying immunotherapies as ineffective, the observed resistance may indicate that both the influence and stimulation on TIME factors cannot meet the minimal requirements for reinvigorating of the immune system. Given that ICPs can serve as a part rather than the opposite side of TIME factors, it may not be necessary to differentiate between ICPs and TIME factors in the future, but rather, they should be targeted together.

Despite of such potential importance and promising prospect, directly targeting TIME regulators in a simple and feasible way is still not easy to achieve, except in a gene-silencing or editing-based strategy. Therefore, whether TIME can be indirectly shaped, and how to put this into practice, have become notable challenges. Over the past 10 years, ever-growing evidence suggests that inducing cancer cell death by activating the immune system is an effective method of anticancer immunotherapy^84–86^. Corresponding to the different stimuli, there are many types of death of cancer cells, such as apoptosis, necroptosis, autophagy, ferroptosis, and pyroptotic cell death^87–89^. From an immunological viewpoint, if cell death is capable to elicit an adaptive immune response toward recognizing and destroying cancer cells via dead cell-associated antigens, this is referred to as ICD^84,88,90^. Correlation analyses showed that ICD mediators were significantly associated with both ICPs and TIME regulators. More importantly, the presented results indicated that ICD exerted an enormous influence on effective T-cell activation. These results suggested that the induction of tumor ICD offers promising prospects for the development of combination immune therapies to broadly and effectively reshape TIME and ultimately reinvigorate the immune system against tumors.

Accumulating preclinical and clinical evidence indicates a conventional chemotherapy-induced immune response to be mainly dependent on ICD induction^34,48,91,92^. Indeed, pharmacological or genetic suppression of ICD largely diminishes the curative effects of anthracyclines-based immunogenic chemotherapy^25,39,40,93–97^. The core phenotypes and mechanisms of immunogenic chemotherapy are very consistent, at least in anthracycline-treated breast cancer, oxaliplatin-treated colorectal cancer, bortezomib-treated multiple myeloma, and imatinib-treated gastrointestinal stromal cancer, despite the slight difference in a tissue-specific fashion or drug-precise pharmacological action^37,98–102^. However, considering the substantial shortcomings in the induction of ICD for cancer therapy, a combination of ICD inducers and ICP-targeted drugs may be an optimal counterplan to help cancer patients in the foreseeable future^8,15,103–105^. Taking immunogenic chemotherapy as an example, ICD is triggered together with a number of undesired immunosuppressive effects, particularly in anthracycline-based therapy^106^. This is why bona fide immune interventions in specific circumstances can improve the chemotherapeutic efficacy to some extent. So far, to neutralize their immunosuppressive effects and maximize the immunostimulatory functions of anticancer drugs, the chemotherapeutic drug gemcitabine has been combined with ipilimumab in preclinical models^107^. Moreover, the BRAF inhibitor dabrafenib in combination with the MEK inhibitor trametinib has also been used with various ICP-targeting agents in an experimental study^108^. More importantly, the CDK inhibitor dinaciclib has recently been reported to induce ICD and enhance anti-PD1-mediated tumor suppression in immunocompetent mice^109^. Furthermore, local chemotherapy can synergize with CTLA-4 inhibition to boost immune response in mice and patients^110^. Intriguingly, a very recent study by Huang et al.^111^ has reported that CALR genetically couples with many ICPs in pancreatic cancer, which is actually the first time to reveal the direct molecular connection between ICD and ICP. Several clinical trials have been launched to test the clinical profile of the synergistic effects of combination therapy with ICD inducers and ICP blockers. This strategy can, at least in principle, not only directly (albeit only partially) kill cancer cells and stimulate short-term immune clearance of the remaining cells, it can also maintain long-lasting immune memory to prevent a recurrence.

A few questions still need to be urgently addressed in the near future: For instance, how should ICD and ICB be scheduled? How can ICD-resistance be avoided? How can the suppressed ICD be restored? How can ICD-induced immunosurveillance be sustained? How can the specificity of ICD inducers be improved? How can new ICD inducers be developed that exert more effects on TIME? Most of these questions should be analyzed in detail for the construction and refining of the conception and system of ICD as well as ICD-based therapy. Consequently, the limitations of targeting TIME regulators can be overcome, which would finally benefit cancer patients by offering timely diagnosis, reasonable treatment, and successful cure in the near future. ICD-mediated TIME regulation is anticipated to open a new research field at the frontier of anticancer immunity^112^.

In conclusion, TIME factors are promising cancer immunotherapeutic targets, and a TIME factor-targeting strategy should be synergized with ICB to fully harness the power of TIME and obtain a maximum clinical benefit for the future cancer immunotherapy. Although direct TIME targeting still remains difficult, ICD inducers may effectively help to reshape TIME and are thus suitable for combination with ICPs inhibitors. Coordinated efforts are required to determine optimal therapeutic combinations and to apply both immune-profiling and genomic-profiling technologies to develop a personalized treatment.

## Methods

### Data collection

To predict the possible outcome of TIME targeting for cancer therapy, potential TIME regulators were collected based on literature and distributed to multiple modules, which include apoptosis (BAK, CASP3, CASP8, and XIAP), necrosis (TNFR1, RIPK3, MLKL, and TRADD), autophagy (ATG16L1, ATG4A, BECN1, and LC3B), metabolism (LDHA, PKM, GLS2, and G6PD), transporter (GLUT1, GLUT4, SLC16A1, and SLC16A3), ATP homeostasis (ATPAF2, CD73, ADA, and PANX1), DAMP (ANXA1, HSP90, PDIA3, and S100A9), growth factor (HGF, HDGF, VEGF, and TGFB), receptor (MET, EGFR, IFNGR, and IFNAR2), ER stress (PERK, EIF2A, XBP1, and ATF6), cell cycle (CDK2, CDK4, CDK6, and CDKN1B), cytokine (CXCL5, CCL22, IL10, and IL18), epigenetics (EZH2, DNMT1, PAD4, and SIRT1), transcriptional factor (MYC, HIF1A, STAT3, and IRF3), tumor antigen (TYRP1, MUC1, DCT, and CA125), and extracellular matrix (MMP1, MMP2, MMP3, and MMP11), respectively. Of note, some selected TIME factors are not only restricted to the tumor microenvironment (TME) but also closely related to tumor burden, which is actually due to the complex interplay and precise regulation between TIME and tumor. For instance, a previous study reported that CDK4, a serine–threonine kinase involved in cell cycle progression and tumor growth, also plays a critical role in antitumor immunity by regulating PD-L1 expression^113^, and thus it was also included in this study. The ICPs investigated in this study included the most well-established PD-L1, PD-L2, CD80, CD86, VTCN1, C10orf54, HHLA2, TNFRSF14, CD155, CD112, CD200, LGALS9, ICOSL, CD137L, CD252, CD70, GITRL, and CD48^111^.

To analyze ICPs and TIME factors-related expressions, prognoses, interactions, associations, or/and correlations in multiple cancers, a total of 31 cancer type-specific datasets in TCGA (http://cancergenome.nih.gov)^114,115^, such as ACC, BLCA, BRCA, CESC, CHOL, COAD, DLBC, ESCA, GBM, HNSC, KICH, KIRC, KIRP, LAML, LGG, LIHC, LUAD, LUSC, OV, PAAD, PCPG, PRAD, READ, SARC, SKCM, STAD, TGCT, THCA, THYM, UCEC, and UCS, were individually collected by the below web servers for multiple bioinformatic analyses^116^. MESO and UVM in TCGA were excluded from the analyses because of data incompleteness. For pancreatic cancer-specific analyses, TCGA and other open-access databases or datasets without overlapping samples were integrated for further bioinformatic analyses. These included four datasets, such as Pancreatic Adenocarcinoma (ICGC, Nature 2012)^117^, Pancreatic Adenocarcinoma (QCMG, Nature 2016)^118^, Pancreatic Adenocarcinoma (TCGA, PanCancer Atlas)^119^, and Pancreatic Cancer (UTSW, Nat. Commun. 2015)^120^. Data from different platforms or laboratories were processed and computed following a standard analysis pipeline.

### GEPIA analysis

GEPIA (http://gepia2.cancer-pku.cn, version 2)^121^ is an open-access online tool for interactive exploration of RNA sequencing expression data of 9736 tumors and 8587 normal samples from the TCGA and the GTEx projects. The gene expression data downloaded from the TCGA and GTEx were recomputed from raw RNA-Seq data by the UCSC Xena project with a uniform pipeline to avoid the data imbalance which can cause inefficiency in various differential analyses. In this study, GEPIA was used to calculate the prognostic indexes of both ICPs and TIME factors. These included the differential profiles of gene expression and patient survival across multiple cancer types, as well as signature correlations in pancreatic cancer. One-way ANOVA was used to analyze the differential expression of ICPs and TIME factors, and genes with |log2FC| values > 1 and *q* values < 0.01 were considered differentially expressed. OS and DFS analyses of ICPs and TIME factors were performed using the Kaplan–Meier method with a 50% (Median) cutoff for both low and high expression groups. Log rank test (the Mantel–Cox test) was used for hypothesis testing, the cox proportional hazards regression model was applied to calculate the hazard ratio, and *p* value < 0.05 was used as a threshold in ranking the results. Spearman correlation analysis was used to analyze the pair-wise gene expression correlations between ICPs and TIME factors, and results with *p* value < 0.01 were selected.

### STRING analysis

STRING (https://string-db.org, version 11.0)^122^ was used to construct protein–protein interactions between ICPs and TIME factors, including physical binding and functional associations. Combined scores were computed by combining the probabilities from different evidence channels, including high-throughput experimental data, mining of databases and literature, and predictions based on genomic context analysis. The combined score ranked from 0 to 1, with 1 indicating the highest possible confidence.

### cBioPortal analysis

cBioPortal for Cancer Genomics (cBioPortal, http://www.cbioportal.org, version v3.2.11)^123^ is an open-access online tool integrating the raw data from large scale genomic projects including but not limited to TCGA and ICGC. The data of gene-level is stored with available clinical information including OS, PFS, DFS, and DSS. In this study, cBioPortal was used for visualization and comparison of genetic alterations of ICPs and TIME factors in pancreatic cancer, as well as alteration-associated contribution to multiple survivors of pancreatic cancer patients. Co-occurrence and mutual exclusivity of genetic alterations between each inquired ICP and TIME factor were determined by log2 odds ratio, *p* value, and *q* value, and results with *q* value < 0.05 were selected. OS, PFS, DFS, and DSS in pancreatic cancer were individually investigated to compare the prognostic differences between altered and unaltered groups, and the Log rank test was used for hypothesis testing.

### TISIDB analysis

TISIDB (http://cis.hku.hk/TISIDB)^124^ is one of the most comprehensive databases for tumor and immune system interactions, which integrates multiple heterogeneous data types, including literature, high-throughput screening data, exome and RNA sequencing data set of patient cohorts with immunotherapy, TCGA, other public databases. These data are integrated into ten categories of information for each gene, such as “function”, “literature”, “high-throughput screening”, “immune therapy”, “tumor-infiltrating lymphocytes (TILs)”, “immunomodulators”, “chemokines”, “subtype”, “clinical”, and “drug”. In this study, TISIDB was used to explore the correlation between the abundance of TILs and the expression of inquired ICPs and TIME factors across multiple cancer types. The immune-related signatures of 28 investigated TIL types were collected according to Charoentong et al.^125^, and the relative abundances of TILs in different cancer types were inferred using gene set variation analysis based on the gene expression profile. Each spearman correlation between the inquired gene and a distinct TIL in an individual cancer type was integrated into the indicated heatmap.

### Reporting summary

Further information on experimental design is available in the Nature Research Reporting Summary linked to this paper.

## Supplementary information

Supplementary Table 1. Co-occurrence between genetic alterations of ICPs and TIME factors in pancreatic cancer

Reporting Summary Checklist

## Data Availability

This pan-cancer analysis study used publicly available datasets. A list of the publicly available datasets analyzed during the study is provided in the figshare repository, as part of the following data record: 10.6084/m9.figshare.13070528^116^. The analysis data files used to generate the figures in the published article are publicly available in the above figshare data record.
